# Predicting Swallowing Recovery in Subacute Stroke Patients via Temporal and Spatial Parameters of Videofluoroscopy

**DOI:** 10.1002/brb3.70104

**Published:** 2024-10-22

**Authors:** Lian Wang, Zhenhai Wei, Wei Xin, Zulin Dou

**Affiliations:** ^1^ Department of Rehabilitation Medicine The Third Affiliated Hospital of Sun Yat‐sen University Guangzhou China

**Keywords:** dysphagia, prognosis, stroke, swallowing, videofluoroscopy

## Abstract

**Purpose:**

The purpose of this study was to identify the temporal and spatial parameters of videofluoroscopic swallowing study (VFSS) that could predict the recovery of swallowing function in subacute stroke patients.

**Methods:**

We included 102 patients who were admitted to the Department of Rehabilitation Medicine between 2019 and 2022. Patients were classified into good and poor prognosis groups according to whether they had restored prestroke swallowing function or were able to consume sufficient nutrition via oral feeding to meet their body's needs. Univariate and multivariate regression analyses were used to identify the predictors. Calibration and discrimination were tested using the Hosmer–Lemeshow test and area under the curve (AUC), respectively.

**Results:**

Of the 102 included patients, 51 had a good prognosis for swallowing function within 6 months of onset. The final multivariate regression model included three significant factors: laryngeal closure duration (LCD) (OR: 0.998; 95% CI: 0.996–0.999; *p* < 0.05), maximum width of the upper esophageal sphincter opening (MWUESO) (OR: 1.251; 95% CI: 1.073–1.458; *p* < 0.05), and pharyngeal residual grade (PRG) (*p* < 0.05). The shorter LCD and larger MWUESO were positive predictors of good swallowing function outcomes, while higher PRG was a negative predictor of good outcomes. The AUC for PRG, MWUESO, and LCD were 0.767 (*p* < 0.05), 0.738 (*p* < 0.05), and 0.681 (*p* < 0.05), respectively.

**Conclusion:**

Identifying prognostic factors for the recovery of swallowing function in patients with poststroke dysphagia is essential for developing treatment strategies. The findings of this study may provide an important reference for developing appropriate therapeutic interventions to promote the recovery of swallowing function in stroke patients.

## Graphical abstract

Predictive role of the temporal and spatial parameters of the VFSS on the prognosis of swallowing function in subacute stroke patients was demonstrated. It was found that LCD, MWUESO, and PRG were three important parameters in predicting the recovery of swallowing function.

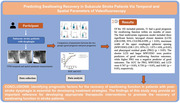

## 1 Introduction

Dysphagia is a common complication of stroke and is closely associated with an increased risk of malnutrition, dehydration, aspiration, and even mortality (Painter, Le Couteur, and Waite 2017; Bath, Lee, and Everton 2018; Simon et al. 2021), which can further impede the functional recovery of patients. Despite a proportion of patients eventually obtaining a satisfactory prognosis spontaneously or with treatment within a short period of time, for some stroke survivors, dysphagia may persist for up to 6 months or even longer (Lee et al. 2020). It is necessary to predict the prognosis of swallowing function, which could help clinicians to make appropriate decisions and optimize clinical care for the patients. For example, in patients at high risk for poor prognosis, alternative feeding modalities (e.g., placement of a percutaneous endoscopic gastrostomy tube) (Kumar et al. 2014) and intensive swallowing rehabilitation training should be considered when necessary. However, an important foundation for predicting recovery of swallowing function is the identification of prognostic factors for swallowing function.

According to previous investigations, the restoration of swallowing function in stroke patients is associated with clinical and demographic factors, such as older age, lower body mass index, higher white blood cell count, bihemispheric infarct, National Institutes of Health Stroke Scale score, intubation, and dysarthria (Kumar et al. 2014; Nakadate et al. 2016). Currently, in addition to the factors mentioned above, efforts are being made to predict the recovery of swallowing function through the kinematic features observed in the videofluoroscopic swallowing study (VFSS), which may be the more accurate predictors.

VFSS can provide visualization of the entire swallowing process and is considered to be a useful tool for describing swallowing function. Previous studies have analyzed the kinematic characteristics of VFSS between tracheotomized and nontracheotomized stroke patients (Seo et al. 2017), as well as investigated the relationship between swallowing kinematics and penetration and aspiration in stroke patients with dysphagia (Seo, Oh, and Han 2016). Recently, a study demonstrated that kinematic analysis of the VFSS is predictive of resumption of oral intake in patients with postoperative oral cancer patients (Okumura et al. 2021). In addition, the kinematic features of VFSS in association with the recovery of swallowing function have been explored in patients with acute stroke (Lee et al. 2021). Also, the kinematic characteristics of VFSS and whether these characteristics can predict the prognosis of swallowing function in subacute stroke patients have drawn the attention of researchers. For instance, Choi et al. (2020) have explored the spatial characteristics of several structures, including the hyoid bone, epiglottis, and vocal cords, and found that epiglottic retroflexion was a critical prognostic factor for swallowing function. However, the predictability of temporal characteristics on the recovery of swallowing function was not analyzed. A previous study investigated the temporal and spatial characteristics of hyoid bone and epiglottis that may be associated with the prognosis of swallowing function in subacute stroke patients with aspiration (Seo, Oh, and Han 2011) but did not include variables that may be associated with aspiration, such as opening of upper esophageal sphincter (UES). In addition, the sample size of the study included only 28 participants, which was relatively small.

VFSS is the gold standard for assessing dysphagia (Lee et al. 2017), and studies have demonstrated that its temporal and spatial parameters can effectively reflect the physiological function of swallowing (Yang et al. 2024; Hanna and Randall 2021; Kim et al. 2023). Furthermore, the studies mentioned above have investigated the predictive role of the temporal and spatial parameters of VFSS on the prognosis of swallowing function; however, there remains a paucity of relevant studies that integrate these parameters for predictive purposes. In particular, there is a lack of studies combining the temporal and spatial parameters of the VFSS to predict the prognosis of swallowing function in subacute stroke patients.

Given the differences in methodology, sample size, and metrics of previous studies, there is a need to further explore the predictive potential of temporal and spatial parameters of VFSS for the recovery of swallowing function in subacute stroke patients. Therefore, we conducted this study to investigate whether the temporal and spatial features of VFSS could be used as predictors for the prognosis of swallowing function in subacute stroke patients with dysphagia.

## 2 Materials and Methods

### 2.1 Study Design and Participants

Ethical approval for this retrospective study was obtained from the Third Affiliated Hospital of Sun Yat‐sen University (no. RG2023‐171‐01). As this was a retrospective study, the informed consent was waived. This study population consisted of consecutive patients with subacute stroke from the Department of Rehabilitation Medicine of the Third Affiliated Hospital of Sun Yat‐sen University between 2019 and 2022. The inclusion criteria were as follows: (1) patients diagnosed with stroke by CT/MRI; (2) swallowing function of the patient was initially assessed by a bedside evaluation by a specialist, and the diagnosis of dysphagia was confirmed by the VFSS examination (Lee et al. 2020); (3) patients with subacute stroke; (4) age > 18 years; (5) dysphagia requiring tube feeding; (6) no dysphagia prior to stroke; and (7) VFSS was performed within 2 weeks of admission. The exclusion criteria were as follows: (1) dysphagia due to other causes (such as Parkinson's disease and nasopharyngeal carcinoma); (2) patients not referred for VFSS; and (3) VFSS images of poor quality that could not be used for analysis.

Data on basic clinical variables were collected by reviewing the patients’ medical records. We recorded the baseline data of patients, including age, sex, stroke type, modified rankin scale (mRS) (Banks and Marotta 2007), and other information, as well as swallowing function evaluation indicators, such as penetration aspiration scale (PAS) score (Alkhuwaiter et al. 2022) and functional oral intake scale (FOIS) score (Crary, Mann, and Groher 2005). FOIS is a seven‐level scale. Specifically, the levels are as follows: Level 1: nothing by mouth; Level 2: tube dependent with minimal attempts of food or liquid; Level 3: tube dependent with consistent oral intake of food or liquid; Level 4: total oral diet of a single consistency; Level 5: total oral diet with multiple consistencies, but requiring a special preparation or compensations; Level 6: total oral diet with multiple consistencies without special preparation but with specific food limitations; and Level 7: total oral diet with no restrictions (Crary, Mann, and Groher 2005). Follow‐up, as a part of patient routine care, of the included patients was performed at 6 months after stroke onset, and the primary research outcome was that they no longer need tube feeding and can intake various textures of food orally to meet their body's nutritional needs.

### 2.2 VFSS Analysis

In the present study, lateral‐view VFSS images at 30 frames/s were acquired using a Lanmage dynamic digital radiography machine (Athena Plus 7500; Shenzhen Lanmage Medical Technology Co. Ltd., Shenzhen, China). VFSS was performed according to a standardized procedure (Logemann 1993). The thickeners (Softia‐S, Nutri. Co. Ltd., Japan) were used to prepare the barium liquid (contrast media: 60% w/v barium sulfate suspension) meeting the standards of Level 1, Level 2, and Level 3 of the International Dysphagia Diet Standardisation Initiative (IDDSI) framework (https://iddsi.org/Framework). In the process, patients were instructed to swallow 3, 5, and 10 mL of thickened and diluted barium liquid in sequence. When the patient experienced significant aspiration or other intolerable events, the examination was stopped. In this study, 5 mL of IDDSI Level 2 liquids were used for analyzing the temporal and spatial parameters of VFSS. The analysis of VFSS was conducted by two trained professionals (M. Dai and C. Li) with at least 5 years of experience who were blinded to the subjects’ information.

#### 2.2.1 Temporal Parameters

Temporal parameters of the VFSS, including hyoid pause time (HPT), pharyngeal transit duration (PTD), and laryngeal closure duration (LCD), were obtained using ImageJ open‐source software.

**FIGURE 1:**
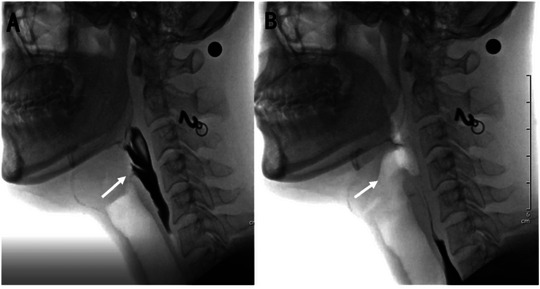
Laryngeal closure duration. (A) The marker (→) indicates onset of laryngeal vestibule closure and (B) re‐opening of the laryngeal vestibule.

LCD (Power et al. 2007) refers to the interval between the first frame that shows contact between the inferior surface of the epiglottis and arytenoids and the first frame that shows the cessation of contact (Figure 1).

PTD (Kendall et al. 2000) refers to the interval from the bolus head at the ramus of the mandible to the bolus head entering the cricopharyngeus.

HPT (Cook et al. 1989) refers to the interval between the arrival of the hyoid bone in the most anterior and superior position and the initiation of fall.

#### 2.2.2 Spatial Parameters

Spatial parameters of VFSS include hyoid bone anterior displacement (HAD), hyoid bone superior displacement (HSD), maximum width of the upper esophageal sphincter opening (Cook et al. 1989) (MWUESO), and pharyngeal residual grade (PRG).

**FIGURE 2:**
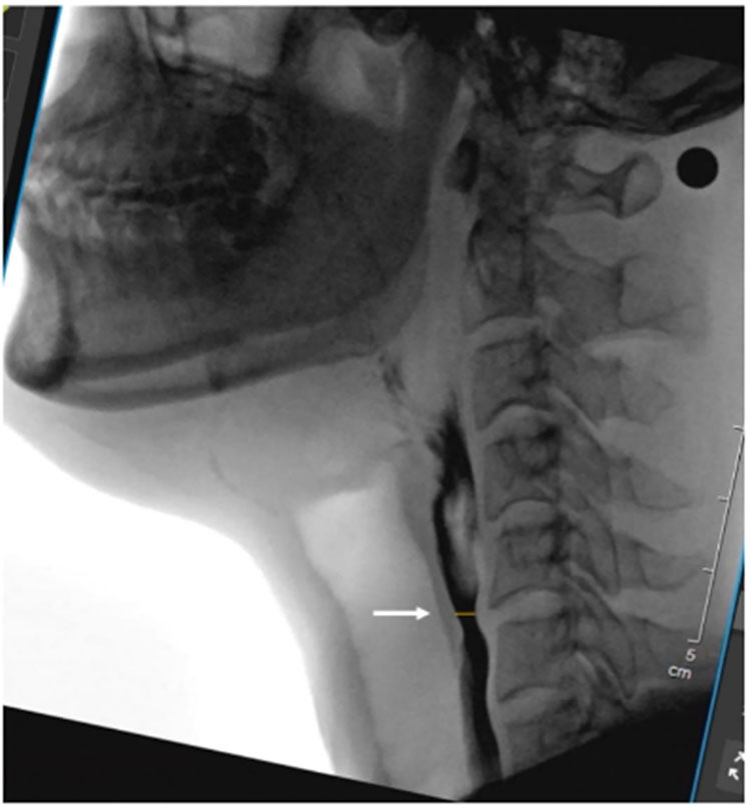
Width of upper esophageal sphincter opening. The marker (→) indicates the maximum width of the upper esophageal sphincter opening.

Measurement of MWUESO as follows: capturing the image when the UES was opened to a maximum in the C4–C6 zone, linking the anterior‐inferior corners of C4 and C6 vertebrae to form a line that makes an acute angle with the vertical axis of the image, and then rotating the image clockwise by the angle (Figure 2).

Hyoid displacement was measured as follows: capturing images at the resting and highest positions of the hyoid bone, respectively; linking the anterior‐inferior corners of C2 and C4 vertebrae to form a line that makes an acute angle with the vertical axis of the image, and then rotating the image clockwise by this angle; marking the coordinates of the hyoid bone and the anterior‐inferior corner of the C4 vertebra in the rotated image as (*X*_R_, *Y*_R_), (*X*_H_, *Y*_H_), (C4*X*_R_, C4*Y*_R_), and (C4*X*_H_, C4*Y*_H_), respectively; and finally, the hyoid bone displacement was calculated according to the following formula:

$$\begin{array}{ccc} \mathrm{HAD} & = & \left(X_{H}-X_{R}\right)-\left(C4X_{H}-C4X_{R}\right),\mathrm{HSD}\\ & = & \left(Y_{H}-Y_{R}\right)-\left(C4Y_{H}-C4Y_{R}\right). \end{array}$$

Both the UESO and hyoid displacement analyses were calibrated using a metal sphere of 8 mm diameter placed at the subject's neck skin as a reference. The PRG scale is divided into four grades, from 0 to 3. Grade 0 represents no residue; Grade 1 represents residue ≤ 10%; Grade 2 represents residue between 10% and 50%; and Grade 3 represents residue ≥ 50% (Han, Paik, and Park 2001).

### 2.3 Measurement of Reliability

We calculated the intraclass correlation coefficient (ICC) for PTD, LCD, HPT, HAD, HSD, PRG, and MWUESO. To assess the reliability of the internal raters, the first rater randomly selected 20% of the subjects and re‐analyzed the above variables one week after the initial rating. The results showed that the ICC values for all variables were > 0.90, indicating a high level of intra‐rater reliability. To assess inter‐rater reliability, another experienced professional randomly selected 20% of the subjects to be analyzed for the above variables. The results showed that the ICC values for all variables were > 0.90, indicating high inter‐rater reliability.

### 2.4 Statistical Analysis

Continuous variables were presented as mean and standard deviation or median with interquartile range, as appropriate. Categorical data were expressed as percentages. The Shapiro–Wilk test was used to determine whether continuous data were normally distributed. Nonnormally distributed continuous variables were tested using the Mann–Whitney U test. Univariate analysis was used to examine the relationship between each factor of interest and the primary outcome variable, and statistically significant variables were entered into multivariate logistic regression models as covariates. The forward selection method was chosen for both univariate and multivariate analyses. All factors identified to be significantly related to the removal of the NGT in the multivariate logistic regression model underwent receiver operating characteristic (ROC) analysis. Calibration and discrimination were tested using the Hosmer–Lemeshow test and area under the curve (AUC), respectively. Statistical significance was set at *p* < 0.05. All statistical analyses were performed using SPSS 26.0 (SPSS Inc., Chicago, IL, USA).

## 3 Results

### 3.1 Patient Characteristics

During the study period, 122 patients with subacute stroke were admitted to the Department of Rehabilitation Medicine, of whom 102 (83.6%) met the inclusion criteria and were analyzed in this study. The baseline characteristics of patients were summarized in Table 1. In total, 51 (50.0%) patients no longer need tube feeding within 6 months after onset and were able to intake various textures of food orally to meet their body's nutritional needs. At follow‐up, patients in the good prognosis group had FOIS scores ranging from 6 to 7, with a median score of 6. These patients no longer required tube feeding. In contrast, patients in the poor prognosis group had FOIS scores ranging from 1 to 4, with a median score of 2. In the poor prognosis group, 38 patients were still dependent on tube feeding, and 13 patients were able to take only a single texture of food orally.

**TABLE 1:** Baseline characteristics.

Characteristics	Good prognosis (*n* = 51)	Poor prognosis (*n* = 51)	*p* value
Age (year), median (IQR)	66 (56, 72)	64 (55, 69)	0.266
Sex, *n* (%)			
Male	36 (70.6)	38 (74.5)	0.657
Lesion laterality, *n* (%)			0.462
Right	20 (40.0)	15 (29.4)	
Left	20 (40.0)	26 (31.4)	
Bilateral	11 (21.6)	10 (19.6)	
Type of stroke, *n* (%)			0.183
Ischemic	34 (66.7)	40 (78.4)	
Hemorrhagic	17 (33.3)	11 (21.6)	
mRS, median (IQR)	4 (3, 4)	4 (3, 4)	0.703
FOIS score, median (IQR)	2 (2, 3)	2 (2, 3)	0.446
PAS, median (IQR)	5 (1, 5)	6 (1, 6)	0.194
Pharyngeal paralysis, *n* (%)	6 (11.8)	10 (19.6)	0.276
Pneumonia, *n* (%)	17 (33.3)	17 (33.3)	1.000
Diabetes mellitus, *n* (%)	19 (37.3)	18 (35.3)	0.837
Tracheotomy, *n* (%)	13 (25.5)	14 (27.5)	0.657
Hemoglobin (g/L), mean ± SD	121.21 ± 14.69	120.66 ± 17.32	0.868
WBC count (× 10^9^/L), mean ± SD	6.98 ± 2.36	7.04 ± 2.42	0.893

Abbreviations: FOIS, functional oral intake scale; IQR, interquartile range; mRS, modified rankin scale; PAS, penetration aspiration scale; SD, standard deviation; WBC, white blood cells.

### 3.2 Predictors for the Recovery of Swallowing Function

**FIGURE 3:**
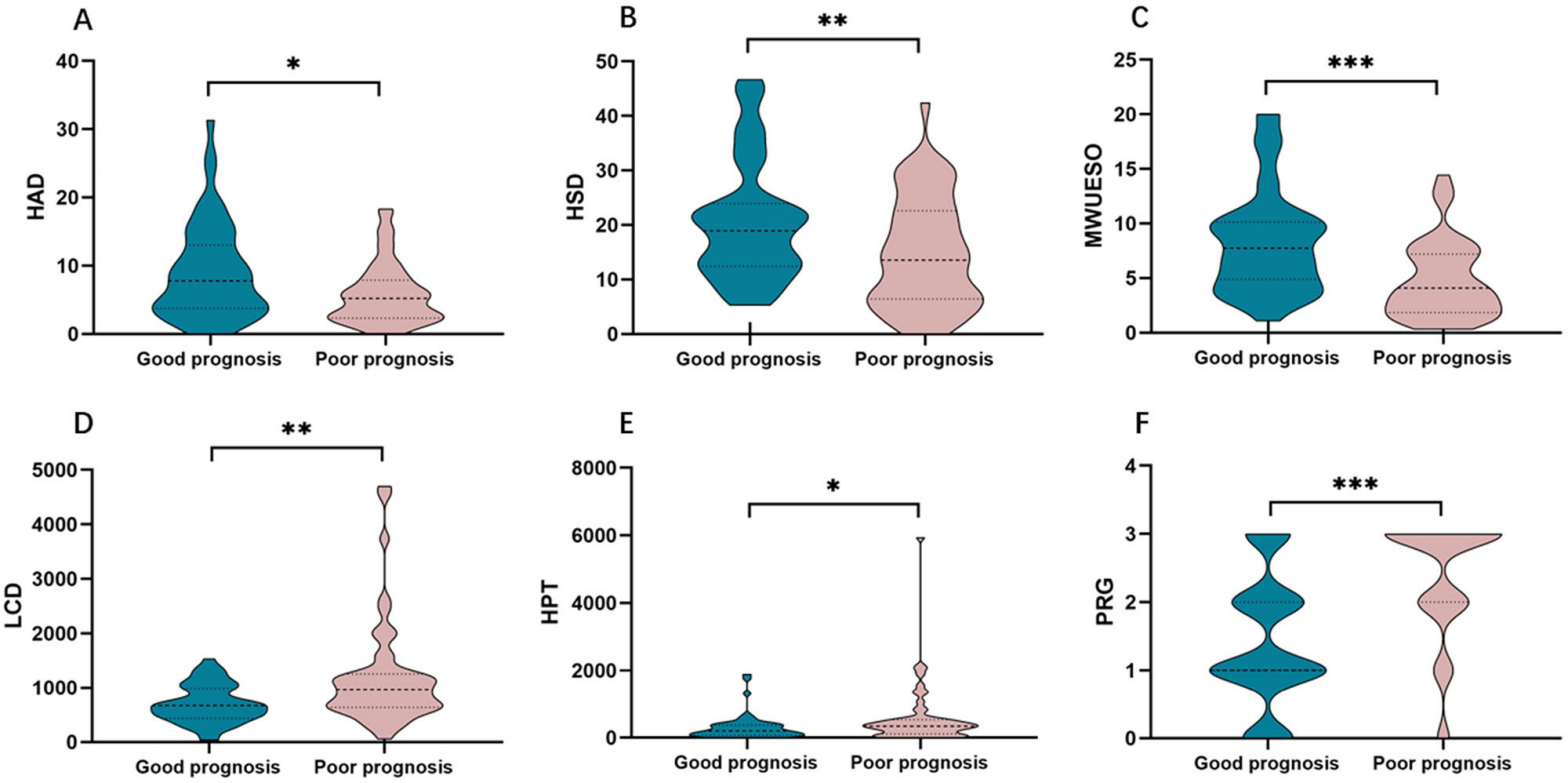
Comparison of parameters between the two groups (good prognosis and poor prognosis). (A–C) Hyoid bone anterior displacement (HAD) (*p* = 0.016), hyoid bone superior displacement (HSD) (*p* = 0.009), and maximum width of the upper esophageal sphincter opening (MWUESO) (*p* < 0.001) in the good prognosis group were significantly larger than those in the poor prognosis group. (D) Laryngeal closure duration (LCD) (p = 0.006) was significantly shorter in the good prognosis group than that in the poor prognosis group. (E) Hyoid pause time (HPT) (*p* = 0.039) was significantly longer in the poor prognosis group than that in the good prognosis group. (F) Pharyngeal residual grade (PRG) (*p* < 0.001) was significantly higher in the poor prognosis group than that in the good prognosis group.

As shown in Figure 3, the differences between the two groups in HAD, HSD, LCD, HPT, PRG, and MWUSEO were statistically significant. HAD (*p* < 0.05), HSD (*p* < 0.05), and MWUESO (*p* < 0.05) were significantly higher in the good prognosis group than in the poor prognosis group. As shown in Figure 3D, LCD was significantly shorter in patients with a good prognosis than patients with a poor prognosis (*p* < 0.05). As shown in Figure 3E, HPT was significantly longer in the poor prognosis group than in the good prognosis group (*p* < 0.05). As shown in Figure 3F, PRG was higher in the poor prognosis group than in the good prognosis group (*p* < 0.05).

**FIGURE 4:**
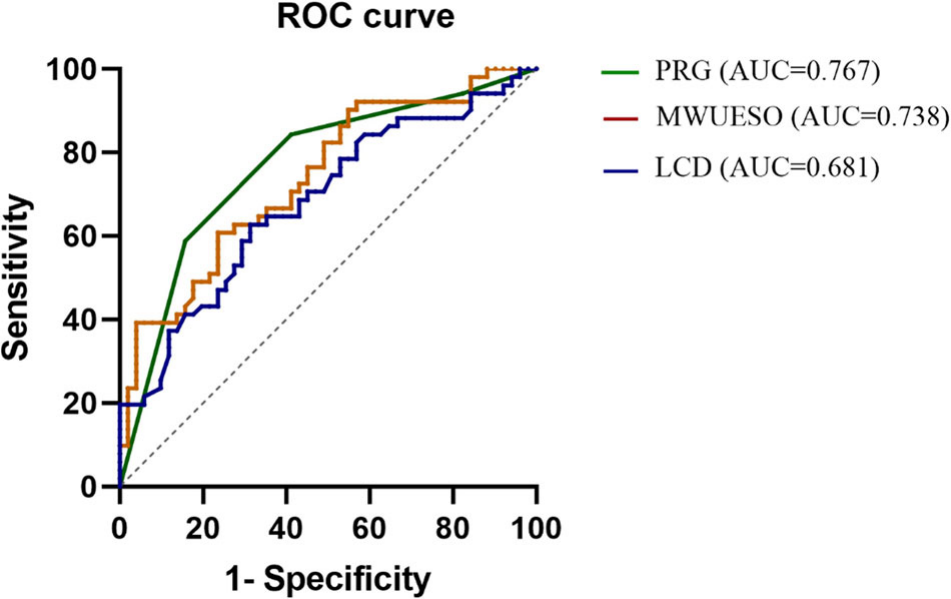
Receiver operating characteristic curve for the multivariable logistic regression model of significant factors predicting the recovery of swallowing function at 6 months after onset in subacute stroke patients. The area under the curve (AUC) in pharyngeal residual grade (PRG) was 0.767 (*p* < 0.001), in upper esophageal sphincter opening (MWUESO) was 0.738 (*p* < 0.001), and in laryngeal closure duration (LCD) was 0.681 (*p* = 0.002), respectively.

These statistically significant variables were included in the univariate regression analysis. In univariate regression analysis, LCD (odds ratio [OR]: 0.998; 95% confidence interval [CI]: 0.997–0.999; *p* < 0.05), HAD (OR: 1.107; 95% CI: 1.025–1.195; *p* < 0.05), HSD (OR: 1.054; 95% CI: 1.013–1.097; *p* < 0.05), PRG (*p* < 0.05), and MWUESO (OR: 1.246; 95% CI: 1.107–1.402; *p* < 0.05) were significant factors (Table 2). The aforementioned variables were included in the multivariate regression model to investigate their effects on the primary outcome variables. In the multivariate model, LCD (OR: 0.998; 95% CI: 0.996–0.999; *p* < 0.05), PRG (*p* < 0.05), and MWUESO (OR: 1.251; 95% CI: 1.073–1.458; *p* < 0.05) were identified as significant factors (Table 3). The AUC in the PRG, MWUESO, and LCD were 0.767 (*p* < 0.05), 0.738 (*p* < 0.05), and 0.681 (*p* < 0.05), respectively (Figure 4).

**TABLE 2:** Univariate logistic regression model for the recovery of swallowing function.

Variable	OR (95% CI)	*p* value
Penetration aspiration scale (PAS) score	0.364 (0.160, 0.830)	0.258
Pharyngeal transit duration (PTD)	1.000 (1.000, 1.000)	0.072
Laryngeal closure duration (LCD)	0.998 (0.997, 0.999)	**0.003** ^∗^
Hyoid pause time (HPT)	0.999 (0.998, 1.000)	0.067
Hyoid bone anterior displacement (HAD)	1.107 (1.025, 1.195)	**0.01** ^∗^
Hyoid bone superior displacement (HSD)	1.054 (1.013, 1.097)	**0.01** ^∗^
Pharyngeal residual grade (PRG)		**< 0.001** ^∗^
Grade 0	Reference	—
Grade 1	1.400 (0.274, 7.149)	0.686
Grade 2	0.333 (0.073, 1.518)	0.156
Grade 3	0.089 (0.019, 0.407)	**0.002** ^∗^
Maximum width of upper esophageal sphincter opening (MWUESO)	1.246 (1.107, 1.402)	**< 0.001** ^∗^

Abbreviations: OR, odds ratio; 95% CI, 95% confidence interval.

**TABLE 3:** Multivariate logistic regression model for the recovery of swallowing function.

Variable	OR (95% CI)	*p* value
Laryngeal closure duration (LCD)	0.998 (0.996, 0.999)	**0.002** ^∗^
Pharyngeal residual grade (PRG)		**0.008** ^∗^
Grade 0	Reference	—
Grade 1	2.027 (0.321, 12.807)	0.453
Grade 2	0.451 (0.086, 2.365)	0.346
Grade 3	0.173 (0.031, 0.963)	**0.045** ^∗^
Maximum width of upper esophageal sphincter opening (MWUESO)	1.251 (1.073, 1.458)	**0.004** ^∗^

Abbreviations: OR, odds ratio; 95% CI, 95% confidence interval.

## 4 Discussion

In this study, we investigated the predictability of kinematic parameters of VFSS for predicting prognosis of swallowing function in subacute stroke patients and identified LCD, PRG, and MWUESO as significant predictors.

Sufficient opening of the UES is the result of precise coordination of multiple events, including hyolaryngeal excursion, neural relaxation, and even bolus propulsive force (Shu et al. 2021). According to the literature, impaired UES opening, which may increase pharyngeal residue and the risk of aspiration, is common in dysphagic patients (Bian et al. 2009). In this study, we found that poorer MWUESO is a negative predictor of prognosis for swallowing function. This finding may shed light on the development of appropriate treatment strategies to promote the recovery of swallowing function. For example, some behavioral interventions, including head lifting exercises (Fujiki et al. 2019), Mendelsohn maneuver (McCullough and Kim 2013), jaw opening, and tongue strength exercises (Wada et al. 2012, Juan et al. 2013), can contribute to the opening of the UES. Note that any impacts on the opening function of UES are multifactorial, and thus the development of treatment strategies needs to be considered from different perspectives.

The importance of laryngeal closure has been robustly supported by previous studies suggesting that laryngeal vestibule closure is the first defense against the entry of swallowed material into the airway (Vose and Humbert 2019). Adequate LCD is critical for effective and safe swallowing (Matsuo and Palmer 2009). With VFSS, it is feasible to visualize the laryngeal closure event and determine the duration of the swallowing process. Bisch et al. (1994) have revealed that patients with dysphagia caused by neurological diseases had significantly shorter LCD than healthy subjects. Park et al. (2010) observed LCD was significantly reduced in stroke patients who aspirated compared with those who did not. These findings indicate that LCD may be abnormal in dysphagic patients and may have an impact on the recovery of swallowing function. Of course, differences in the design of each study, patient heterogeneity, and other factors may affect the results of trials. For example, a previous study showed that LCD was not significantly altered in stroke patients compared with healthy controls. To the best of our knowledge, studies on LCD predicting the recovery of swallowing function in subacute stroke have not been reported. Interestingly, we found that reduced LCD was identified as a significant predictor of good swallowing function outcomes. In the good prognosis group, the reduced LCD may result from the smooth passage of bolus through the esophagus, i.e., a shortened time for the entire swallow. In this study, although there was no significant difference in PTD between the two groups, the poor prognosis group had a longer pharyngeal transit time than the good prognosis group, which may affect LCD. In addition, the HPT was significantly shorter in the good prognosis group than those in the poor prognosis group, which may also be a factor affecting LCD. Thus, to further investigate the factors influencing LCD, a multicenter study with a larger sample size is still necessary. Additionally, from a therapeutic perspective, previous studies have shown that bolus properties can affect the duration of physiological events in swallowing (Power et al. 2007, Bisch et al. 1994, Logemann et al. 2002). Thus, the effect of different bolus consistencies and volumes on the duration of physiological events may be potentially therapeutic dysphagia. Combined with previous research, the findings of this study may help to develop targeted treatment programs.

An increase in pharyngeal residue reflects an underlying feature of impaired or incomplete pharyngeal transport (Perlman, Booth, and Grayhack 1994; Dodds, Logemann, and Stewart 1990). Horner et al. (1991) indicated that the degree of pharyngeal residual strongly correlates with the development of aspiration. Similarly, Eisenhuber et al. (2002) showed that aspiration was a frequent finding in dysphagic patients with pharyngeal residues and that the risk of aspiration significantly increased with the amount of pharyngeal residue. Worse still, aspiration may lead to the risk of more serious complications, such as aspiration pneumonia. Therefore, severe pharyngeal residues may contribute to poor prognosis of swallowing function. In line with these findings, our results showed that the severe PRG has predictability for poorer prognosis of swallowing function.

Hyoid displacement plays a crucial role in safe and effective swallowing by contributing to the sealing of the larynx and opening of the UES. Therefore, it is an important physiological event to measure. Notably, we found that hyoid displacement was the variable of significance; however, it was not included in the final multivariate model during the analysis. Similar results were reported by Okumura et al. (2021), who reported that hyoid bone displacement was not predictive of prognosis of swallowing function in patients with oral cancer. In contrast, Lee et al. (2021) found that a reduction in the horizontal anterior displacement of the hyoid bone during swallowing was potentially a kinematic feature indicative of a poor swallowing prognosis in patients with PSD. Regarding this, we think it may be that the hyoid body is a rather small anatomical feature in the whole VFSS image (Donohue et al. 2021), which may lead to deviation when marking its coordinates. Moreover, its location varies depending on age, sex, and other factors (Loth et al. 2015). These findings may account for inconsistent results. Therefore, the predictability of hyoid bone displacement on swallowing outcomes in stroke patients still deserves further exploration.

Our research serves as a significant supplement to the existing studies on VFSS parameters for predicting the prognosis of swallowing function in subacute stroke patients (Choi et al. 2020, Seo, Oh, and Han 2011), and our findings may provide insights for further research and clinical applications in this field. However, the definition and measurement of swallowing events were not completely consistent across studies. Furthermore, the measurement of the temporal and spatial parameters of VFSS relies on the skills and experience of the professionals, which may lead to inter‐rater variability in the measurements. All these factors may have an impact on the study findings. Therefore, it is important to carefully consider these potential factors when translating research findings into clinical practice.

This study has some limitations. First, our findings are restricted to subacute stroke patients and cannot be extended to all stroke populations; thus, caution is still warranted when interpreting our results. Second, although 3, 5, and 10 mL thickened and diluted barium liquid were used in the VFSS examination, only 5 mL was analyzed in this study. Since VFSS kinematic parameters such as UES opening may be affected by bolus size, it is warranted to further analyze the prediction of VFSS kinematic parameters of different bolus sizes on the prognosis of swallowing function in the following study. Third, the present study lacks kinematic analysis of VFSS in the oral phase, such as oral transit time. Therefore, further studies are needed to elucidate the relationship between other kinematic parameters of the VFSS and the prognosis of swallowing function in stroke population. Finally, the prognosis of swallowing function is related to a variety of factors, including nutrition, interventions, and care of the patient's family; however, the factors included in this study are relatively limited, and the most basic clinical information should be considered.

## 5 Conclusion

In summary, the swallowing characteristics of the VFSS, including LCD, PRG, and MWUESO, are of great significance in predicting the recovery of swallowing function in patients with subacute stroke at 6 months of onset. Clinicians should be aware of these important factors, as they can be used to develop appropriate treatment strategies for patients with PSD. Our results support that the temporal and spatial analysis of VFSS can be considered a valuable predictor of the recovery outcome of swallowing function in subacute stroke patients. However, factors associated with the resumption of oral intake varied significantly depending on patient characteristics and other variables. Therefore, more factors should be considered in future research based on VFSS to predict the recovery of swallowing function after stroke.

## Author Contributions

**Lian Wang**: writing–original draft, methodology, writing–review and editing, formal analysis, conceptualization, software. **Zhenhai Wei**: formal analysis, methodology. **Wei Xin**: formal analysis, methodology. **Zulin Dou**: supervision, writing–review and editing, conceptualization.

## Conflicts of Interest

The authors declare no conflicts of interest.

### Peer Review

The peer review history for this article is available at https://publons.com/publon/10.1002/brb3.70104.

## Data Availability

The data that support the findings of this study are available from the corresponding author upon reasonable request.
